# Supplementary figures and images for: Establishment and quality evaluation of a glioma biobank in Beijing Tiantan Hospital
Source: PeerJ. 2018 Mar 13;6:e4450. doi: 10.7717/peerj.4450 (PMC5855883; doi:10.7717/peerj.4450)

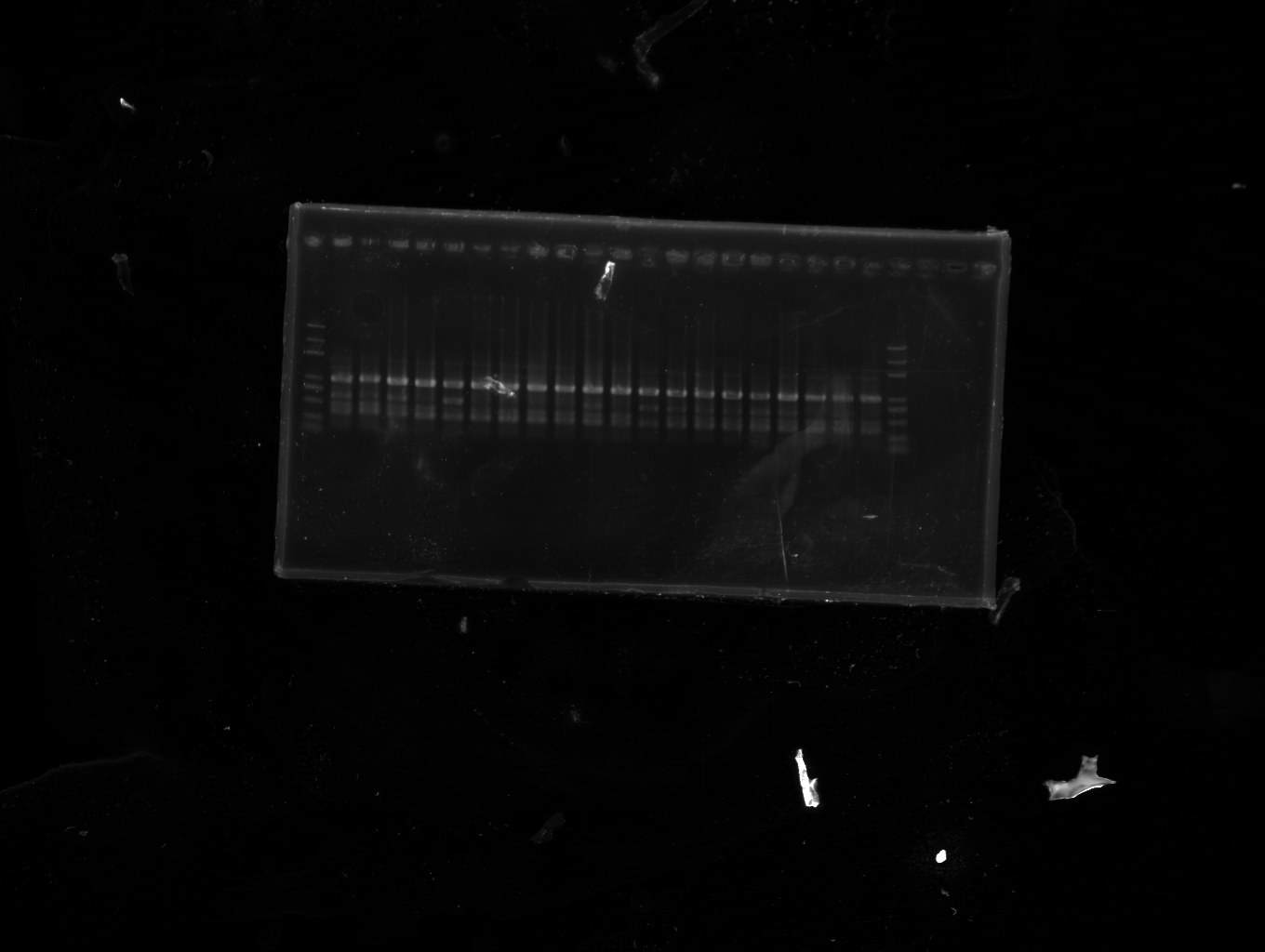

Supplement: Supplemental Information 1 [file peerj-06-4450-s001.zip › year2011-Primer III.tif]

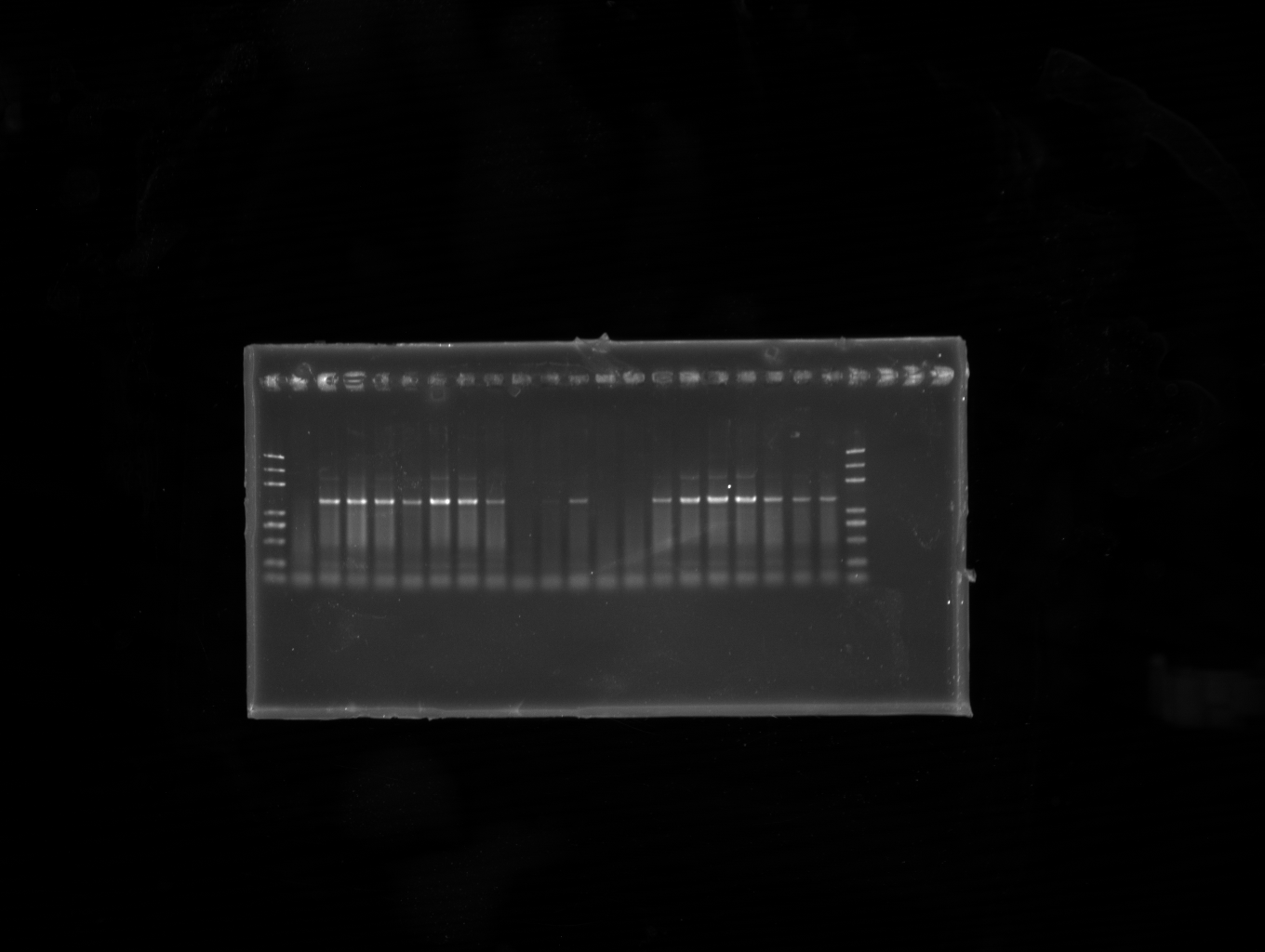

Supplement: Supplemental Information 1 [file peerj-06-4450-s001.zip › year2011-Primer IV.tif]

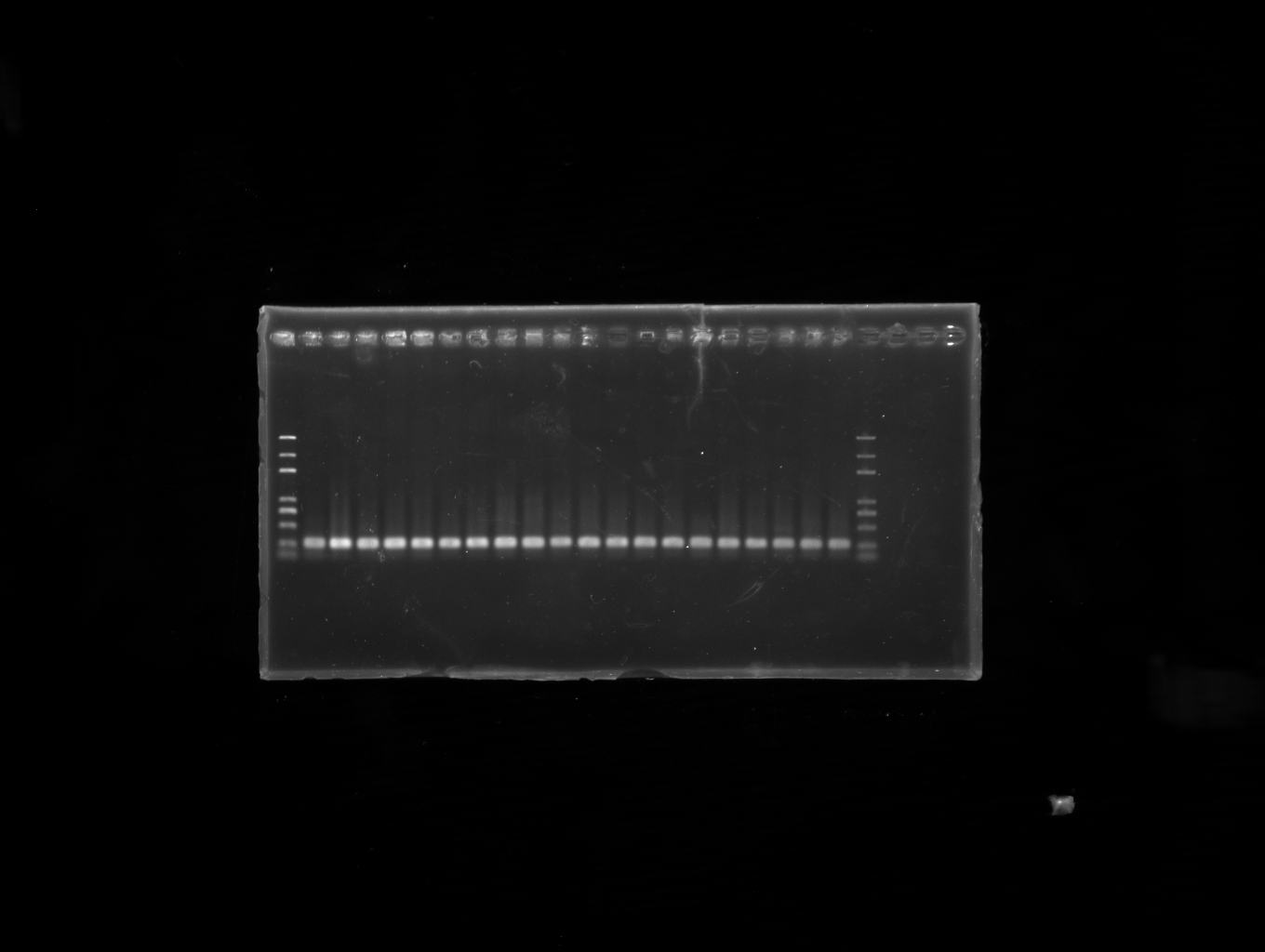

Supplement: Supplemental Information 1 [file peerj-06-4450-s001.zip › year2012-Primer I.tif]

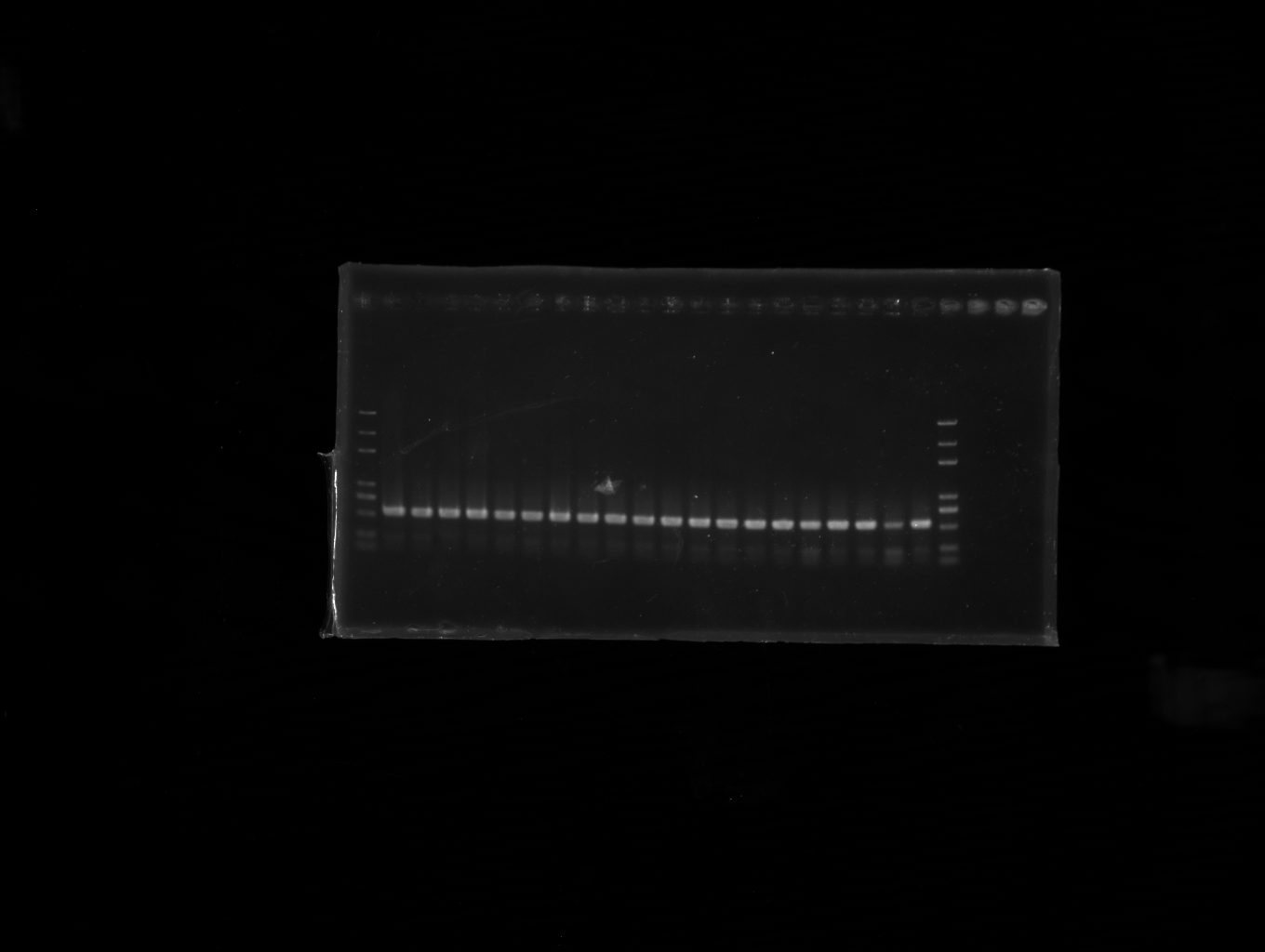

Supplement: Supplemental Information 1 [file peerj-06-4450-s001.zip › year2012-Primer II.tif]

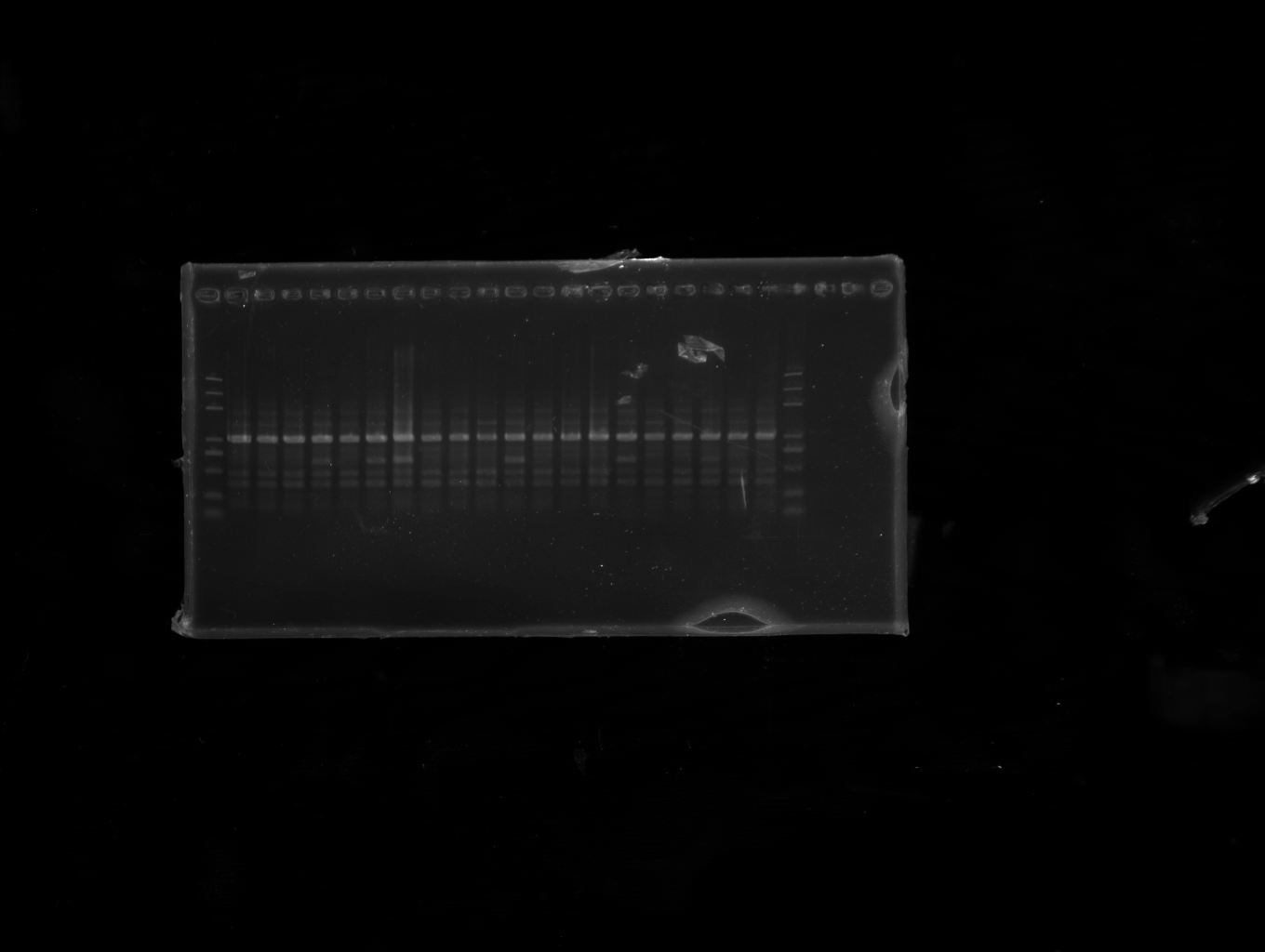

Supplement: Supplemental Information 1 [file peerj-06-4450-s001.zip › year2012-Primer III.tif]

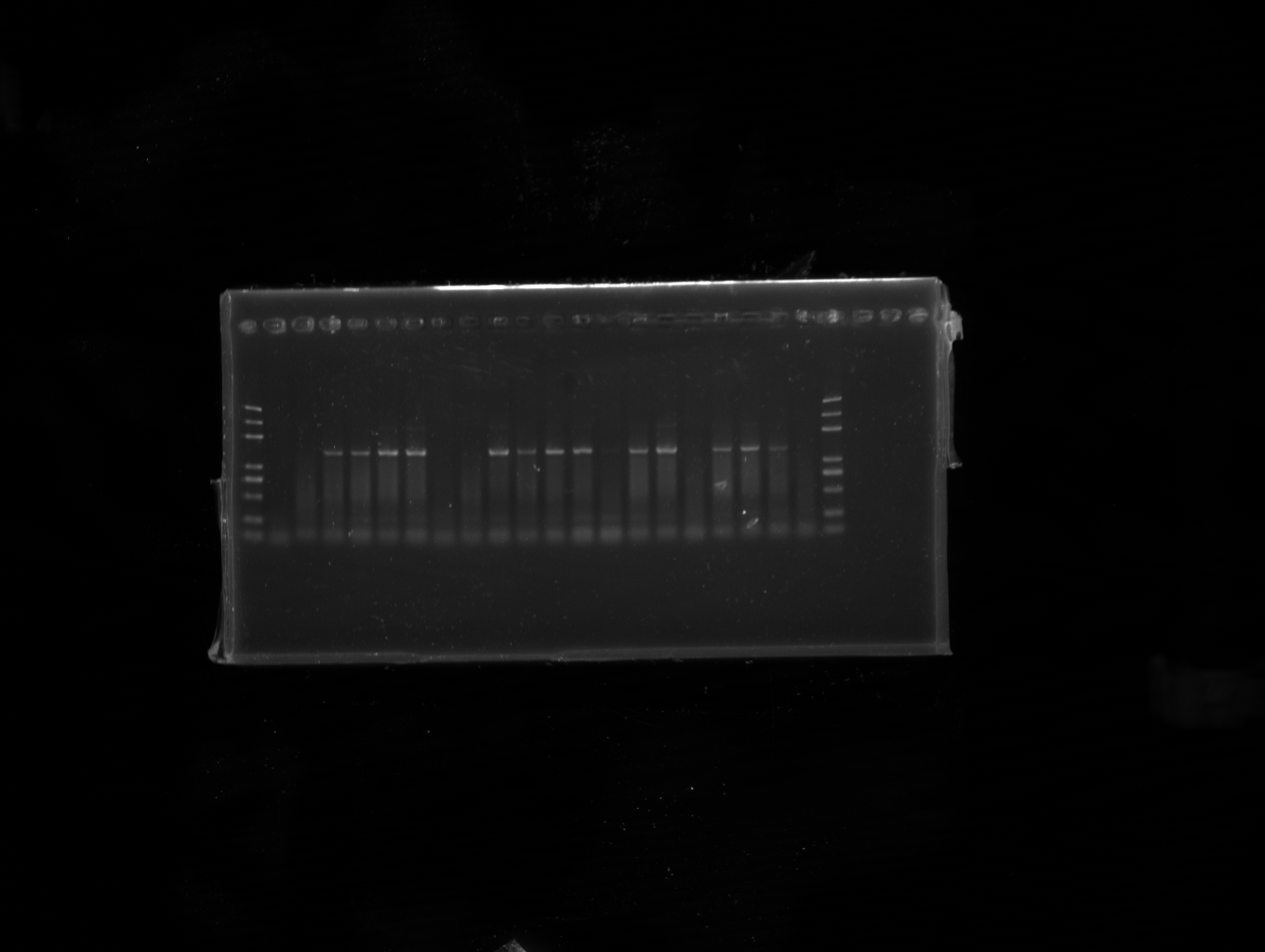

Supplement: Supplemental Information 1 [file peerj-06-4450-s001.zip › year2012-Primer IV.tif]

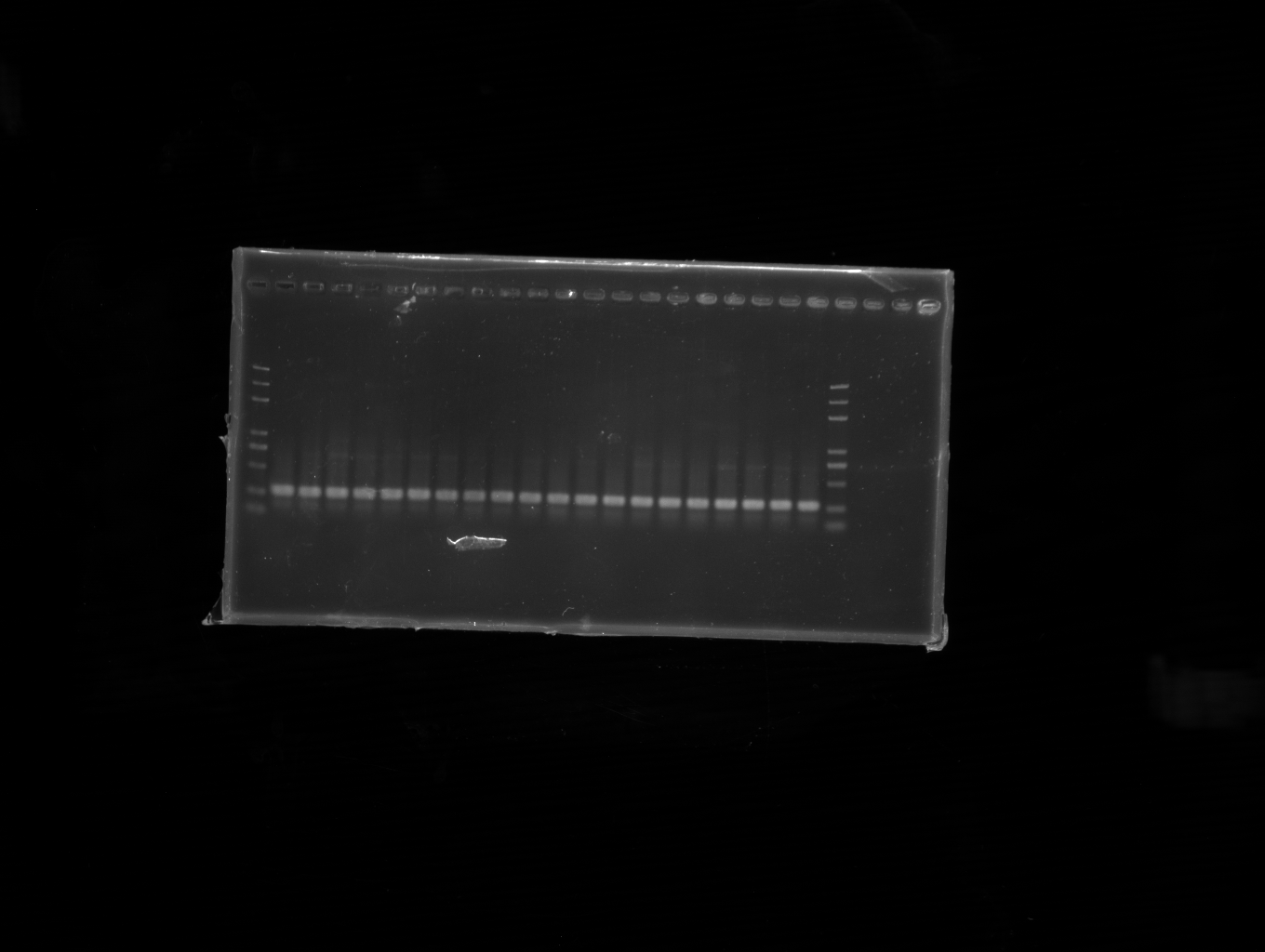

Supplement: Supplemental Information 1 [file peerj-06-4450-s001.zip › year2013-Primer I.tif]

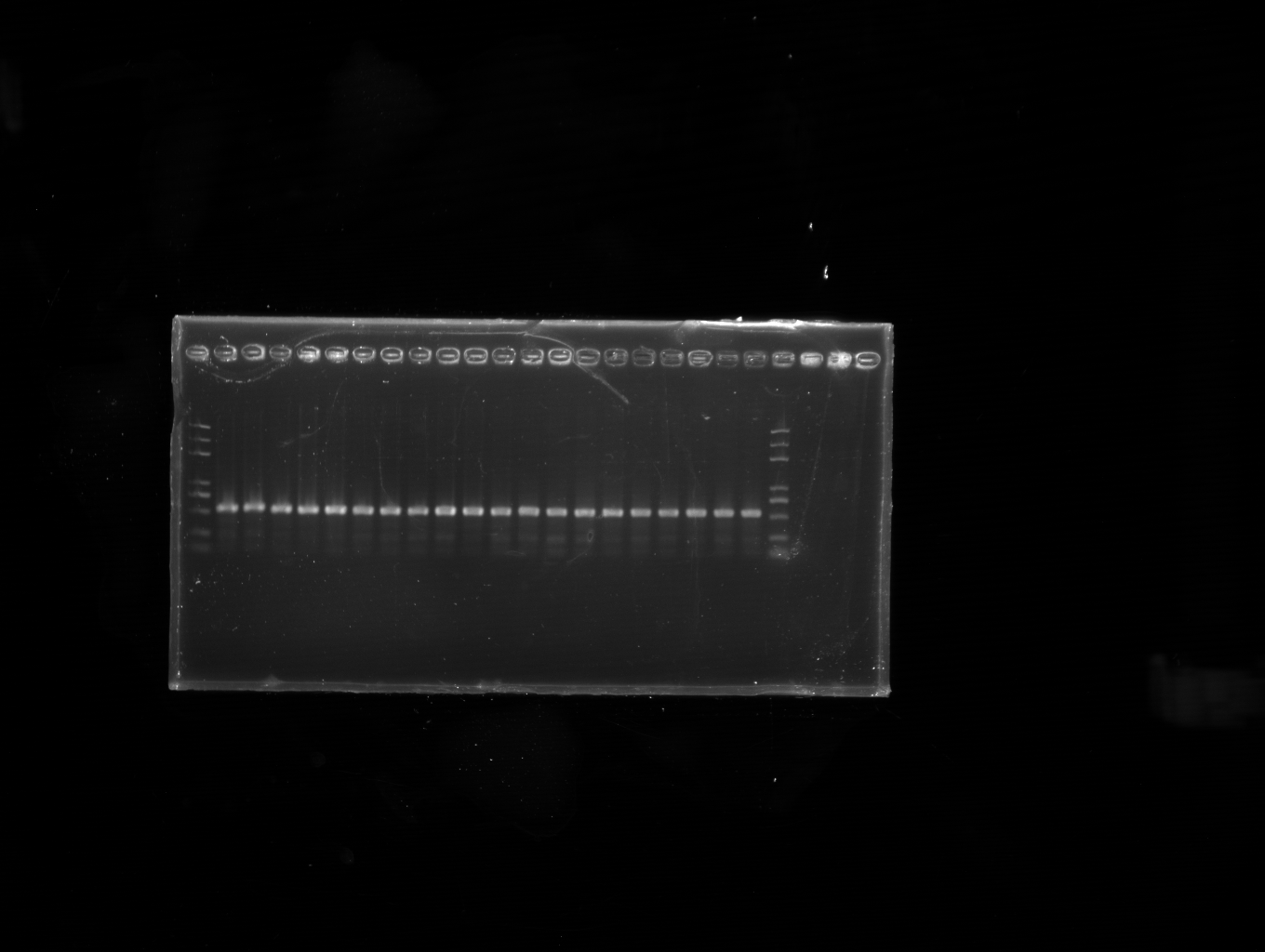

Supplement: Supplemental Information 1 [file peerj-06-4450-s001.zip › year2013-Primer II.tif]

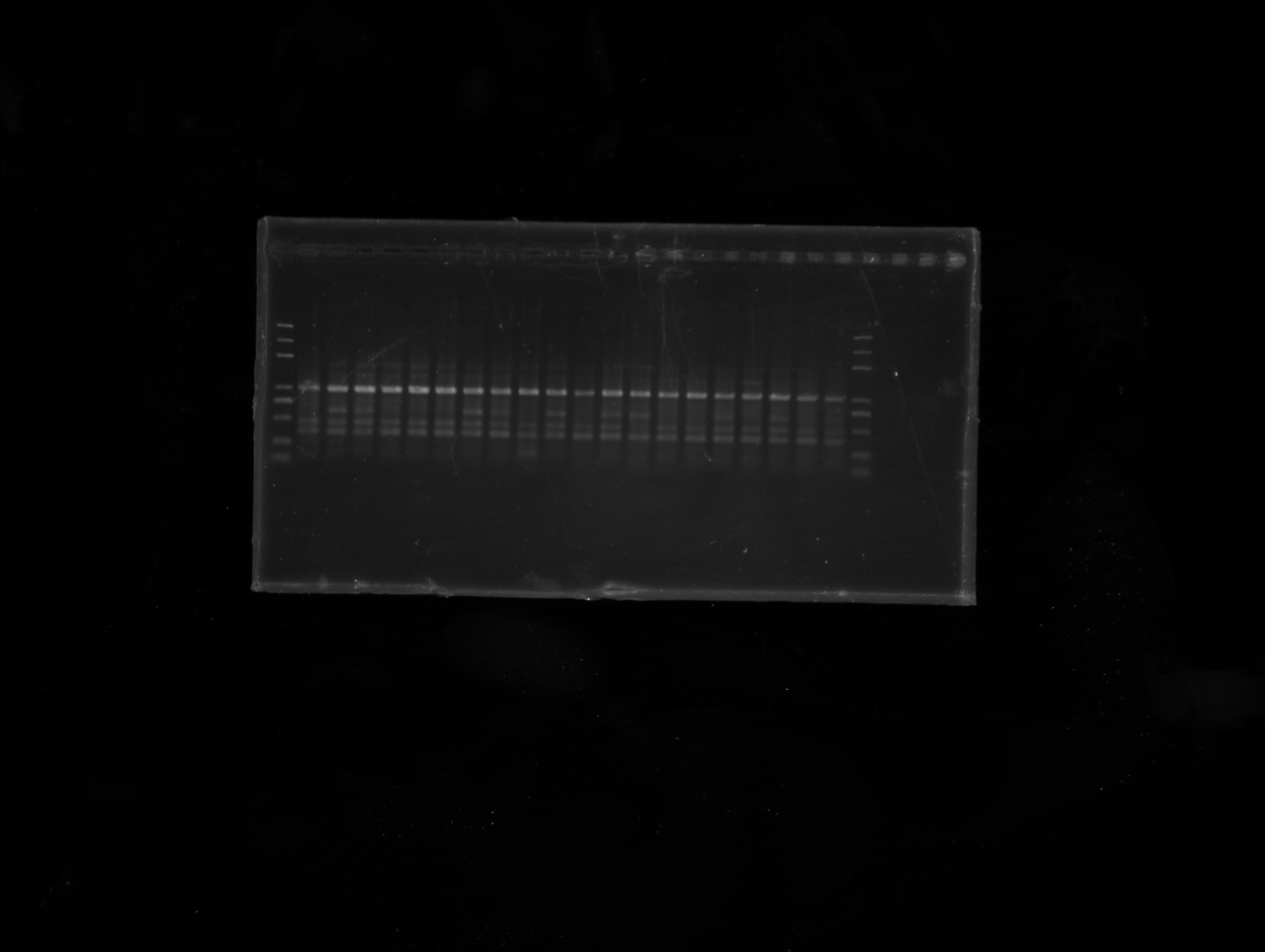

Supplement: Supplemental Information 1 [file peerj-06-4450-s001.zip › year2013-Primer III.tif]

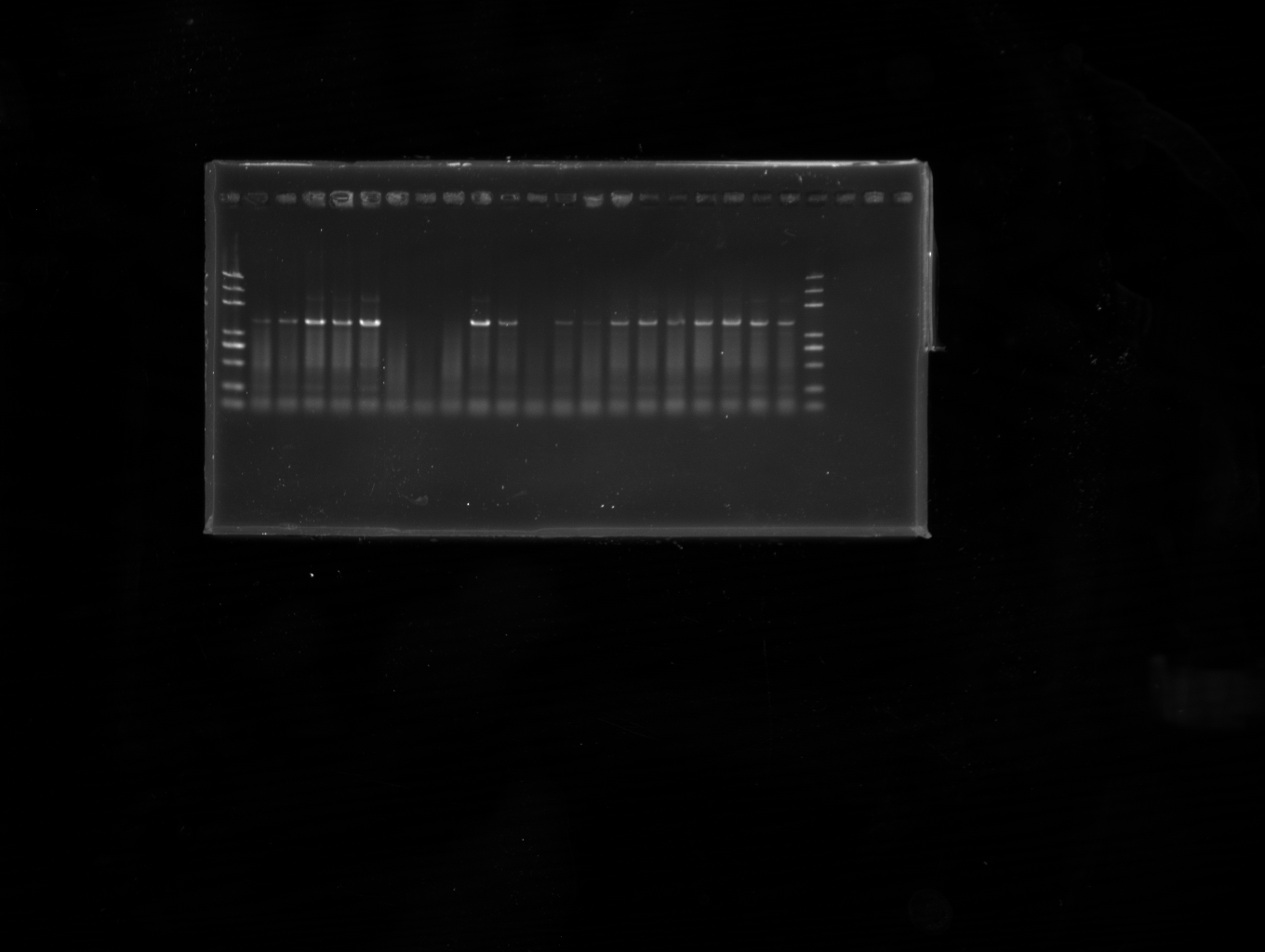

Supplement: Supplemental Information 1 [file peerj-06-4450-s001.zip › year2013-Primer IV.tif]

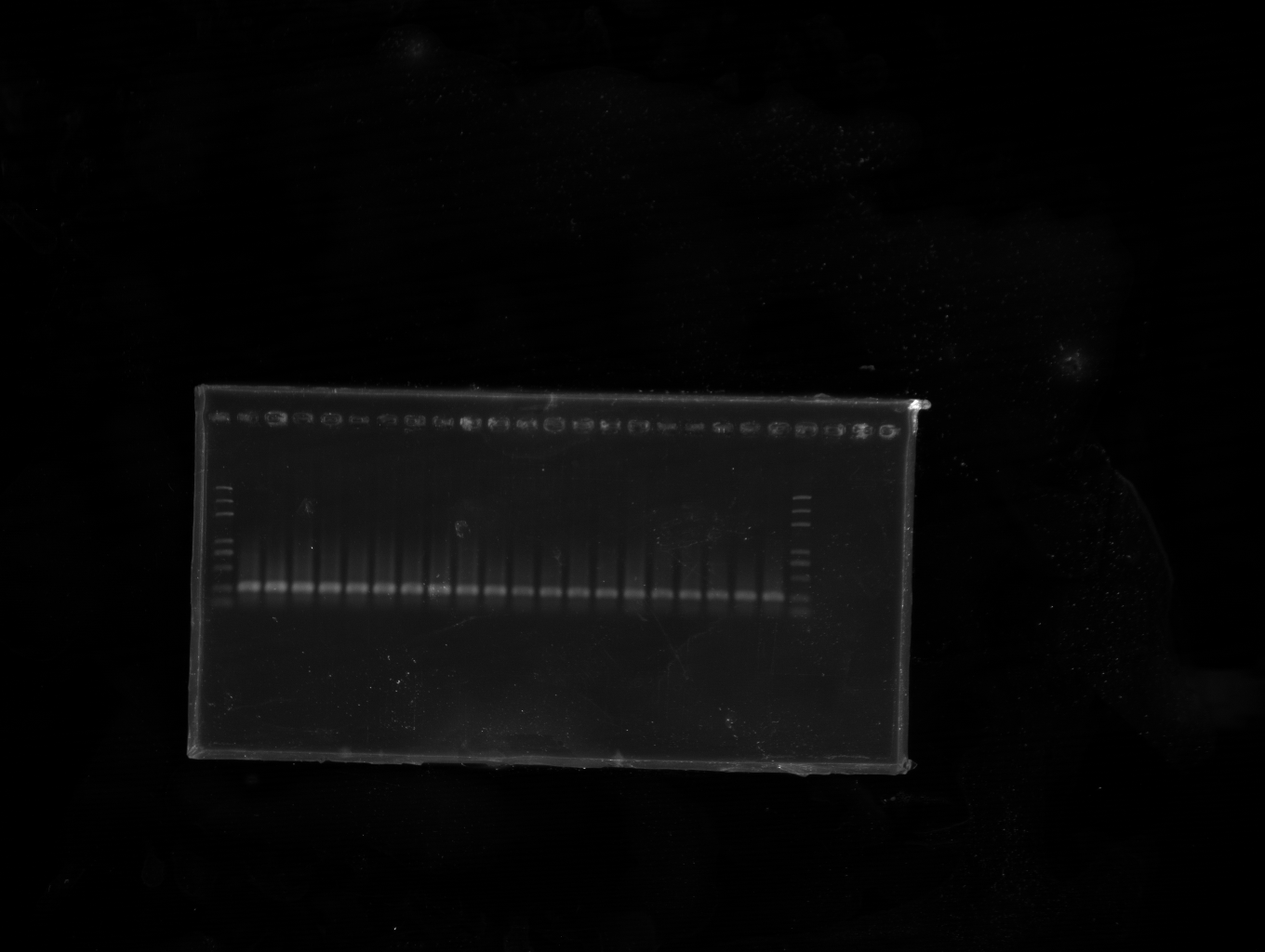

Supplement: Supplemental Information 1 [file peerj-06-4450-s001.zip › year2014-Primer I.tif]

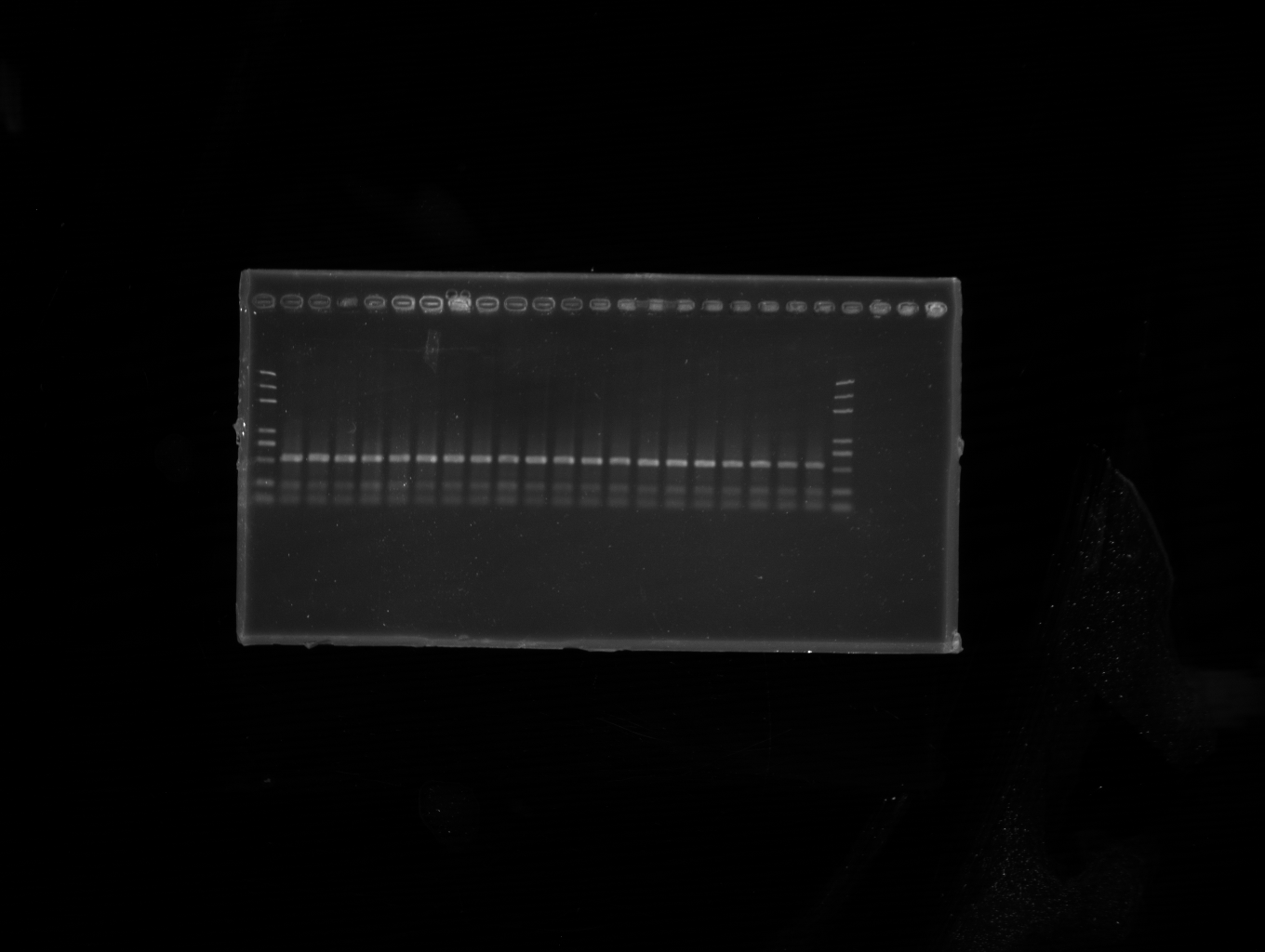

Supplement: Supplemental Information 1 [file peerj-06-4450-s001.zip › year2014-Primer II.tif]

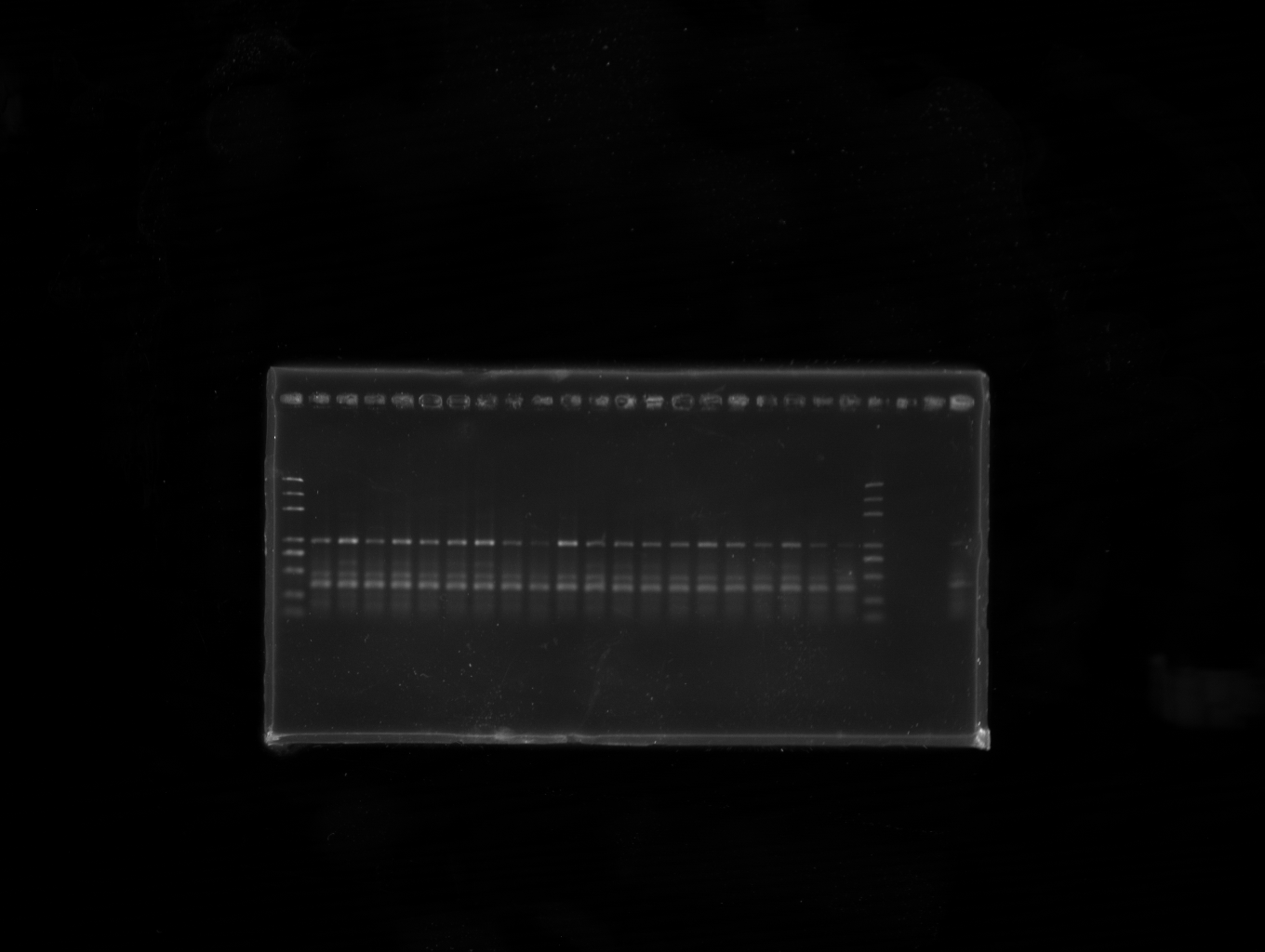

Supplement: Supplemental Information 1 [file peerj-06-4450-s001.zip › year2014-Primer III.tif]

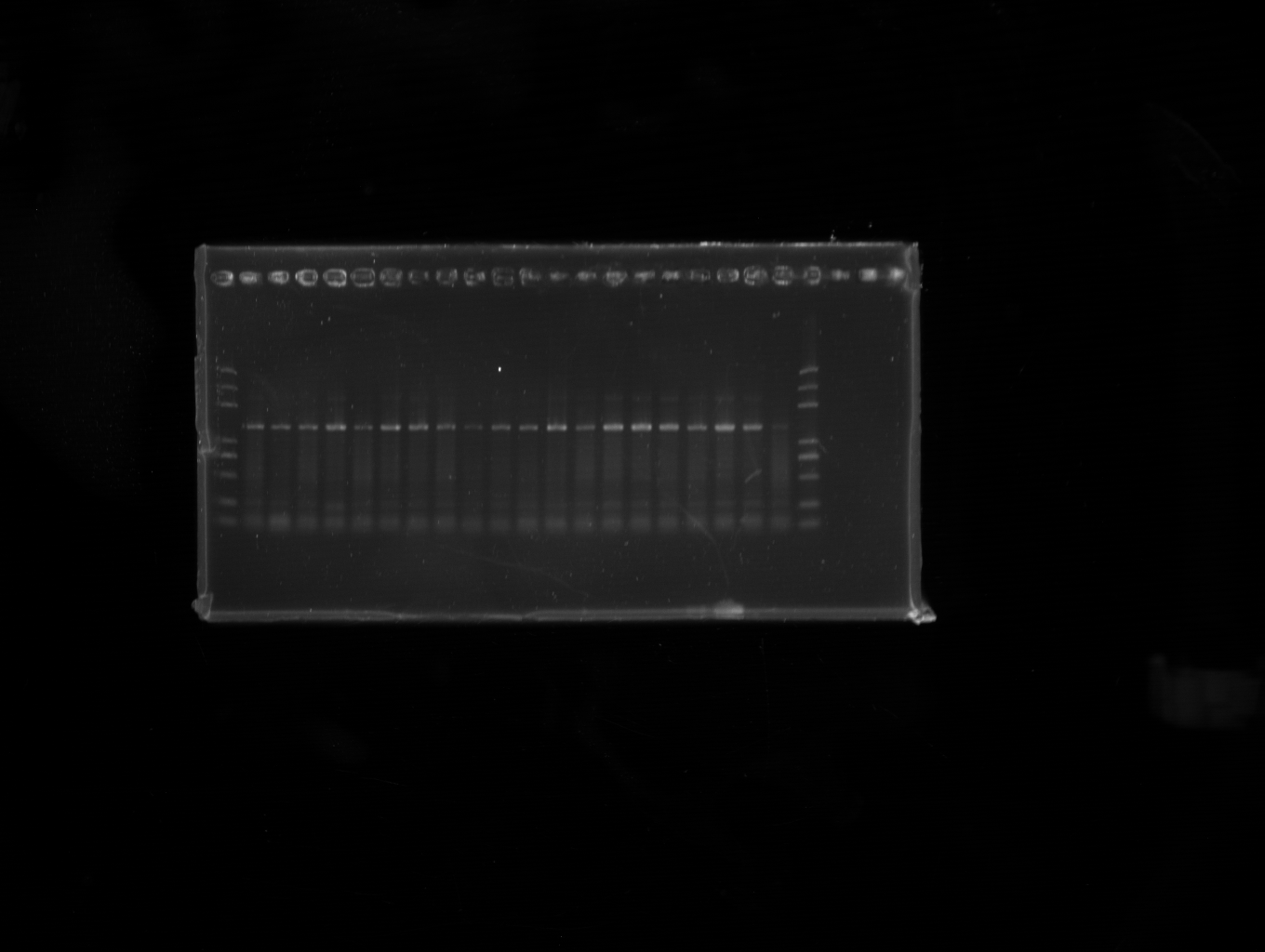

Supplement: Supplemental Information 1 [file peerj-06-4450-s001.zip › year2014-Primer IV.tif]

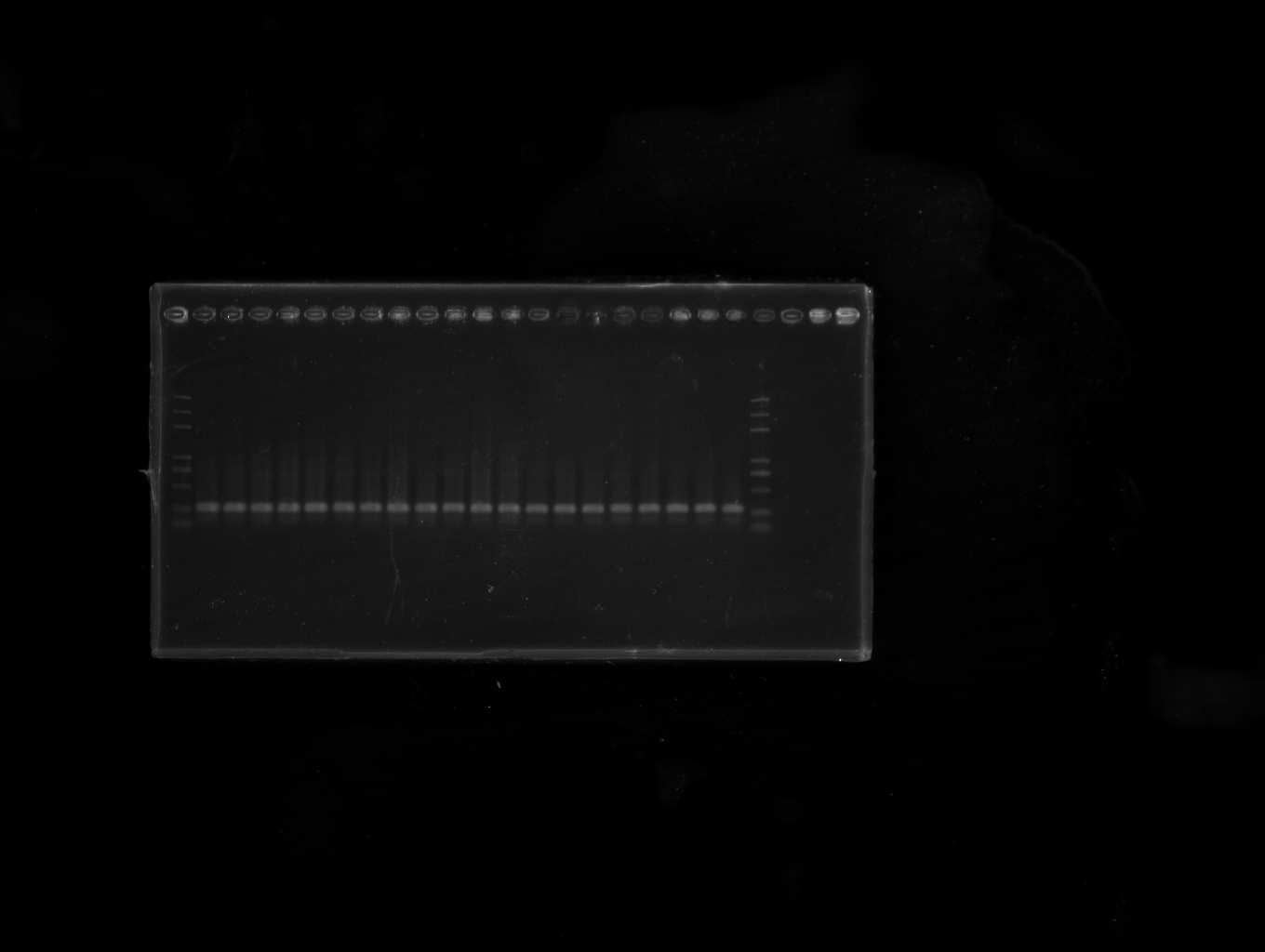

Supplement: Supplemental Information 1 [file peerj-06-4450-s001.zip › year2015-Primer I.tif]

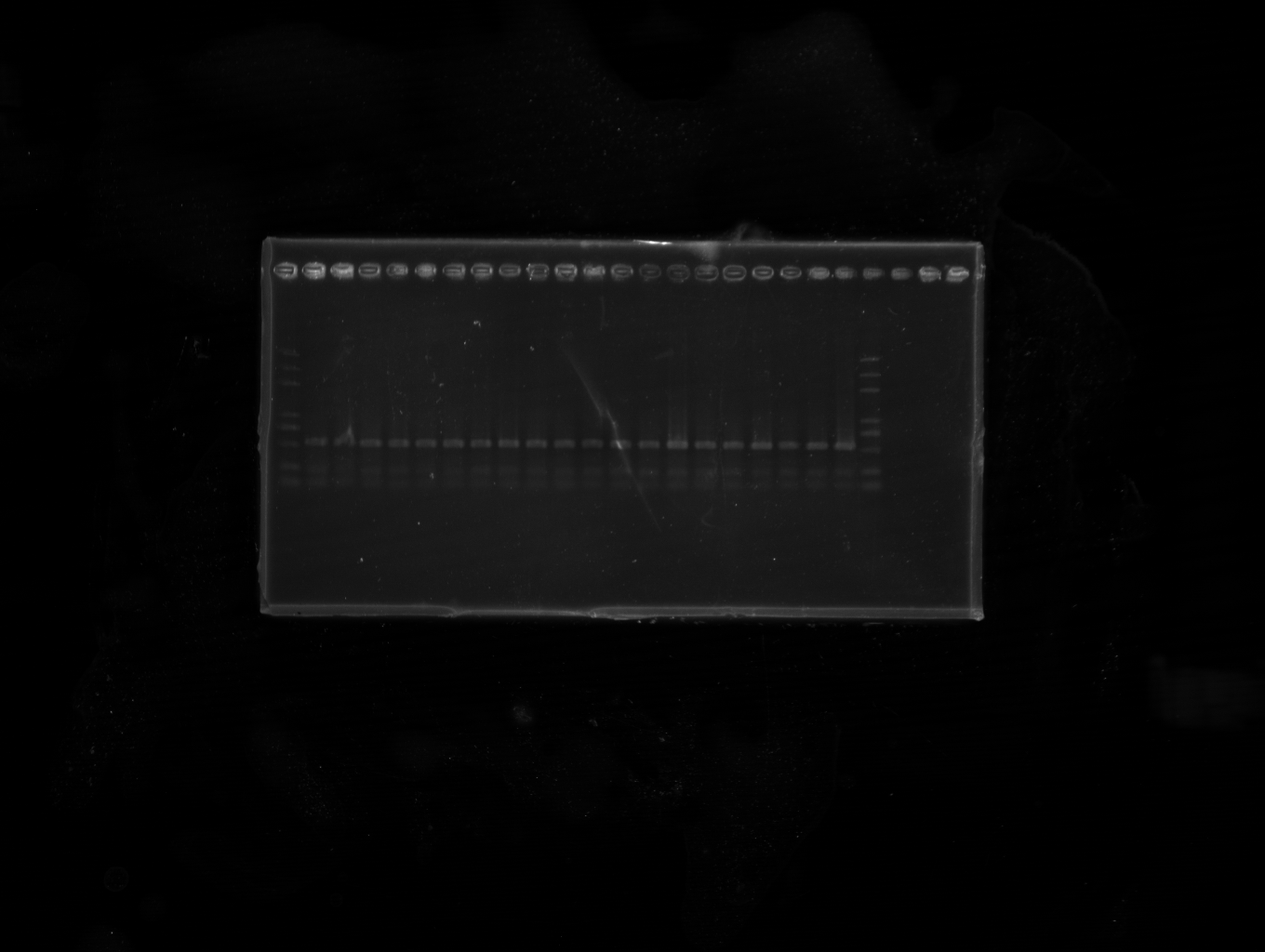

Supplement: Supplemental Information 1 [file peerj-06-4450-s001.zip › year2015-Primer II.tif]

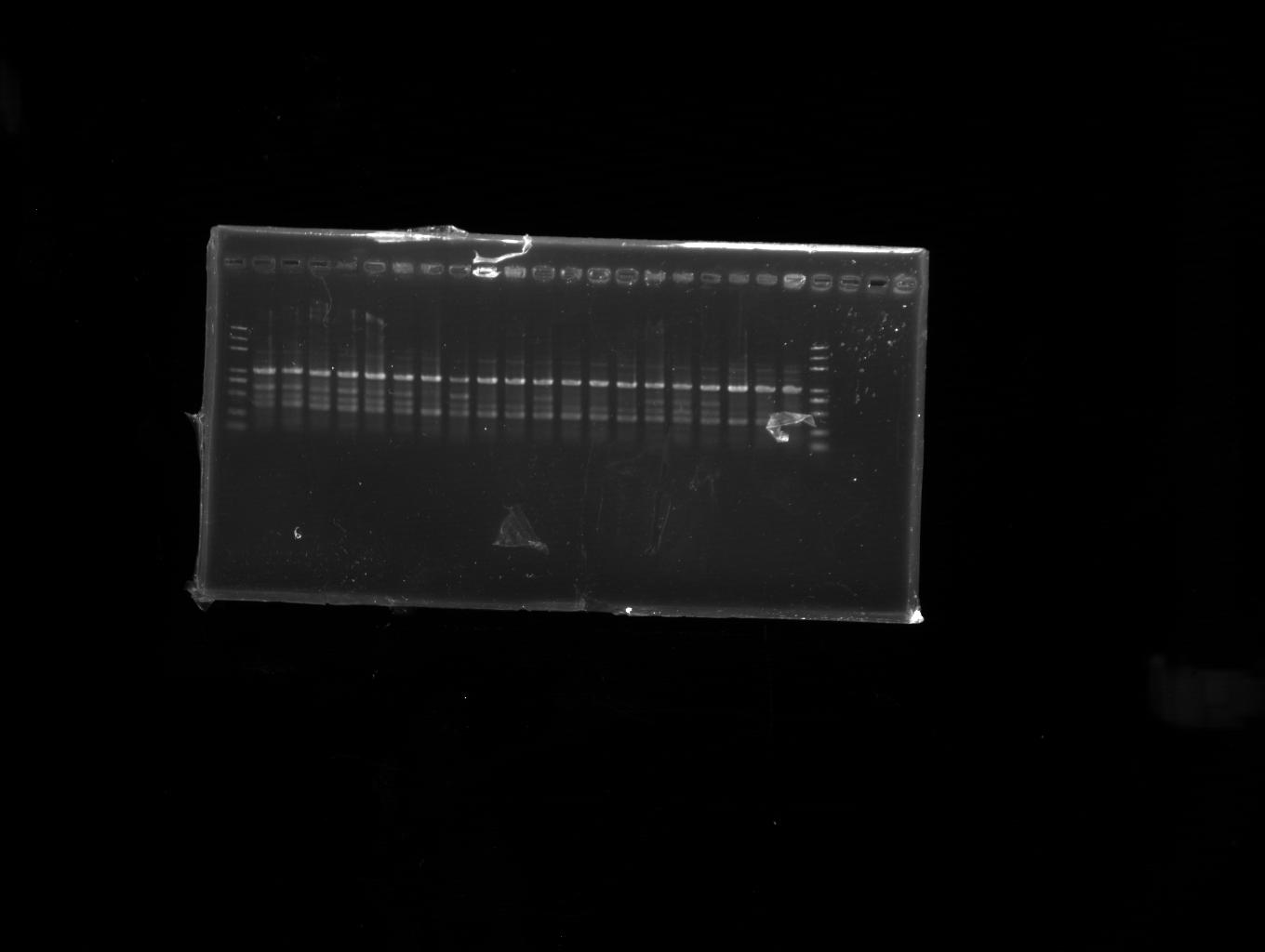

Supplement: Supplemental Information 1 [file peerj-06-4450-s001.zip › year2015-Primer III.tif]

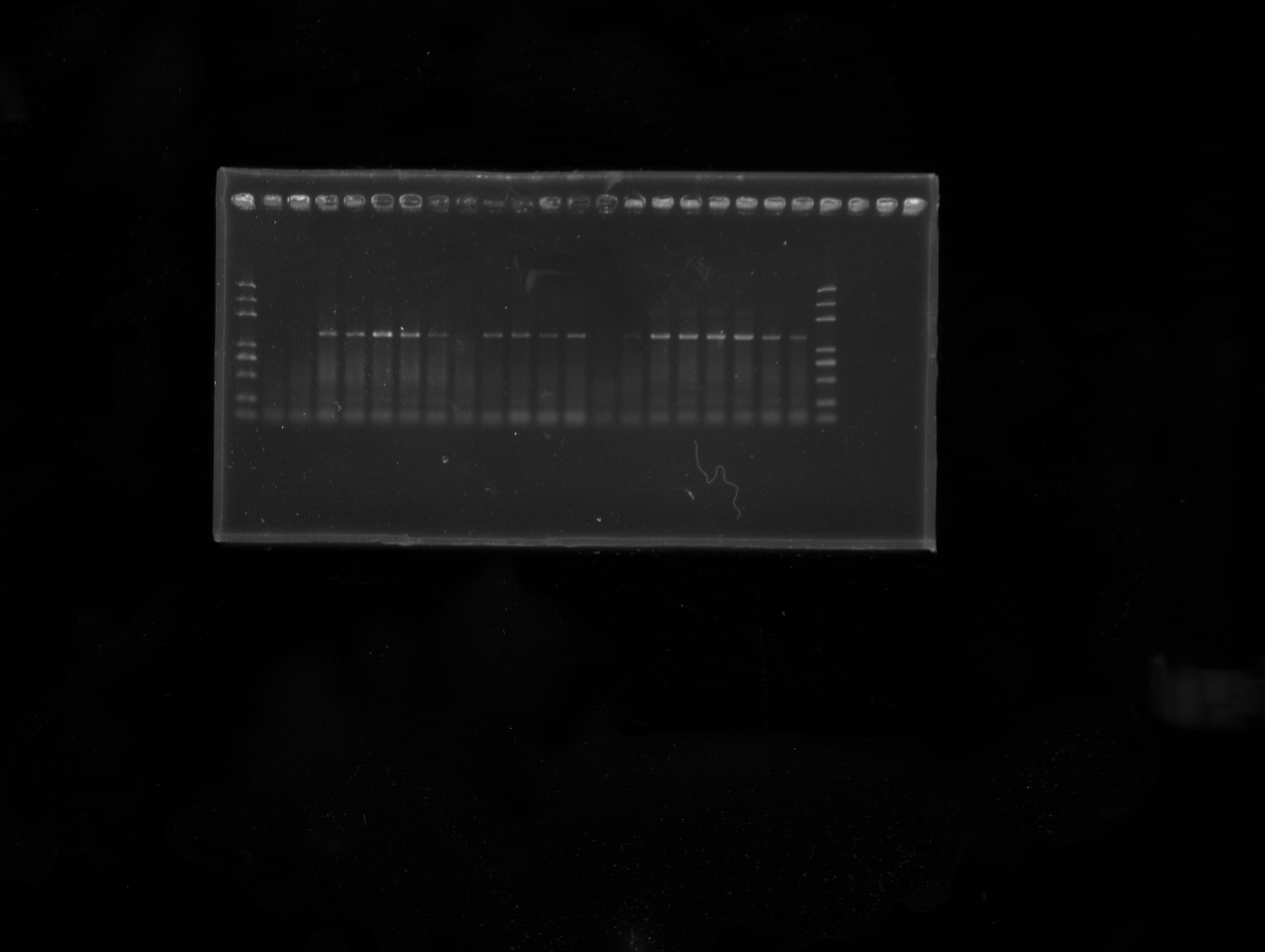

Supplement: Supplemental Information 1 [file peerj-06-4450-s001.zip › year2015-Primer IV.tif]

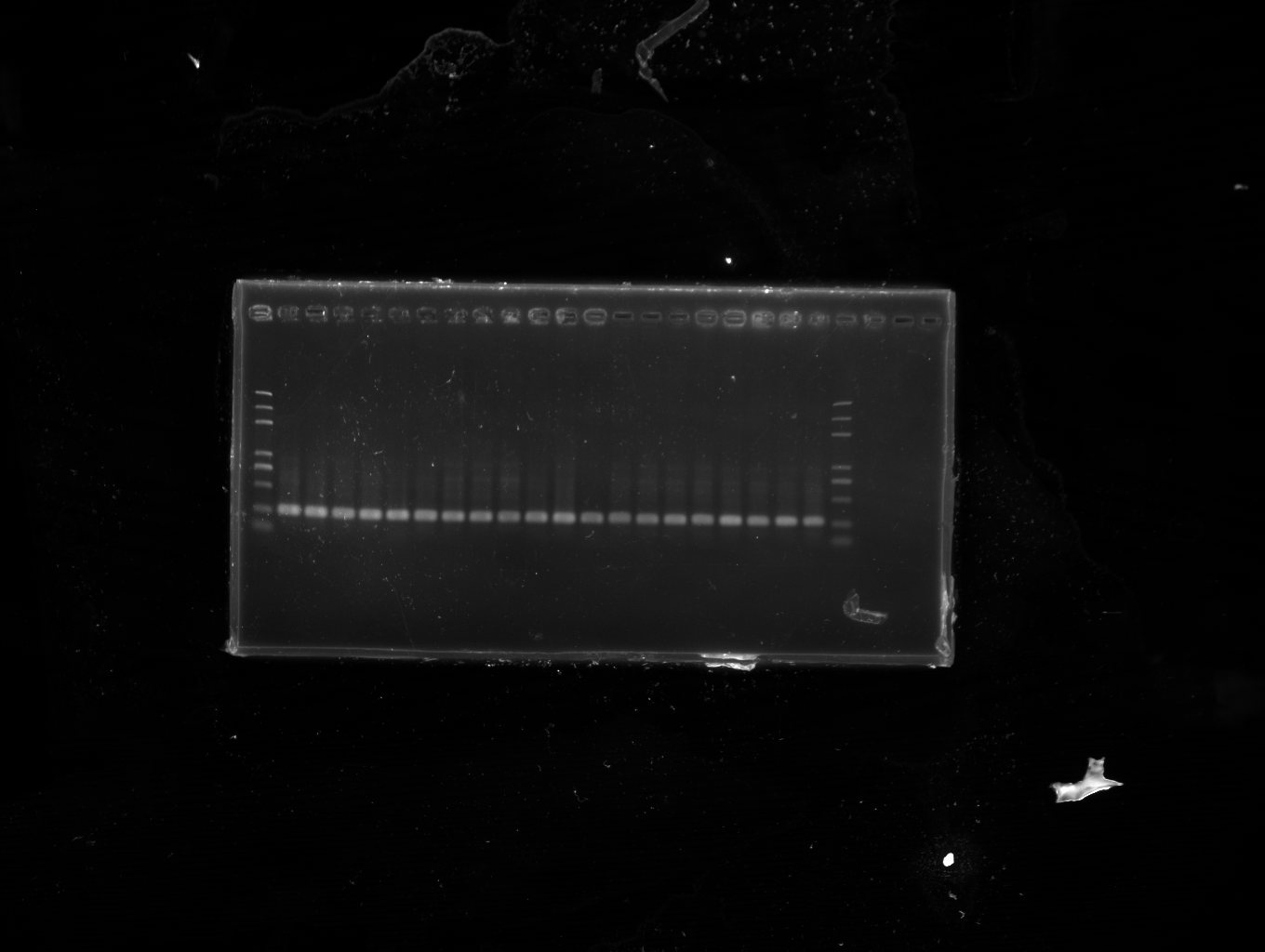

Supplement: Supplemental Information 1 [file peerj-06-4450-s001.zip › year2011-Primer I.tif]

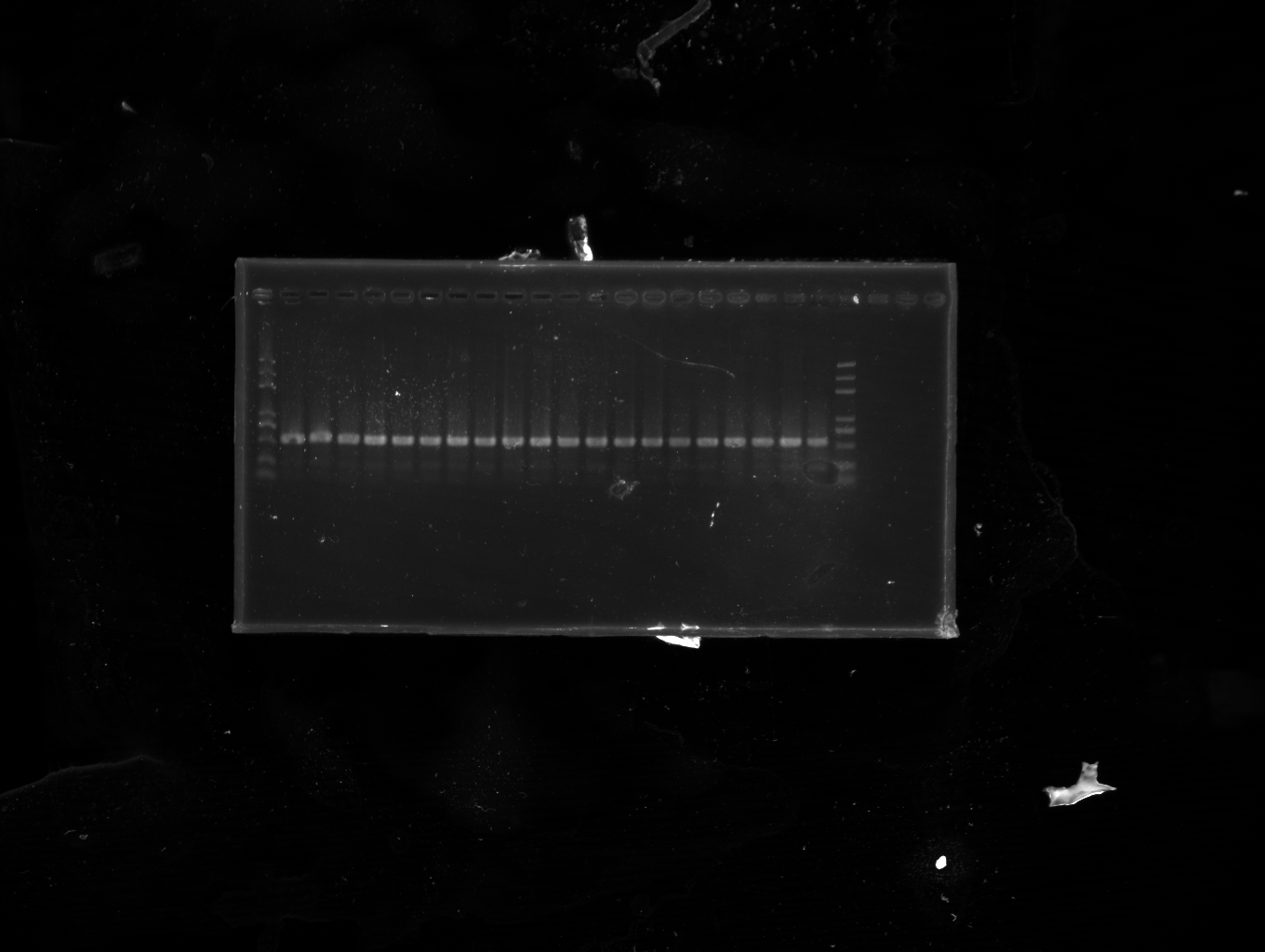

Supplement: Supplemental Information 1 [file peerj-06-4450-s001.zip › year2011-Primer II.tif]
